# A 10-m annual grazing intensity dataset in 2015–2021 for the largest temperate meadow steppe in China

**DOI:** 10.1038/s41597-024-03017-5

**Published:** 2024-02-10

**Authors:** Chuchen Chang, Jie Wang, Yanbo Zhao, Tianyu Cai, Jilin Yang, Geli Zhang, Xiaocui Wu, Munkhdulam Otgonbayar, Xiangming Xiao, Xiaoping Xin, Yingjun Zhang

**Affiliations:** 1 College of Grassland Science and Technology, China Agricultural University, Beijing, 100193 China; 2 Hubei Key Laboratory of Regional Ecology and Environmental Change, China University of Geosciences, Wuhan, 430074 China; 3 College of Land Science and Technology, China Agricultural University, Beijing, 100193 China; 4 Department of Natural Resources and Environmental Sciences, University of Illinois at Urbana-Champaign, Urbana, IL 61801 USA; 5 Division of Physical Geography and Environmental Research, Institute of Geography and Geoecology, Mongolian Academy of Sciences, Ulaanbaatar, 15170 Mongolia; 6 Department of Microbiology and Plant Biology, Center for Earth Observation and Modeling, University of Oklahoma, Norman, OK 73019 USA; 7 National Field Scientific Observation and Research Station of Hulunbuir Grassland Ecosystem in Inner Mongolia, Institute of Agricultural Resources and Regional Planning, Chinese Academy of Agricultural Sciences, Beijing, 100081 China

**Keywords:** Grassland ecology, Ecological modelling

## Abstract

Mapping grazing intensity (GI) using satellites is crucial for developing adaptive utilization strategies according to grassland conditions. Here we developed a monitoring framework based on a paired sampling strategy and the classification probability of random forest algorithm to produce annual grazing probability (GP) and GI maps at 10-m spatial resolution from 2015 to 2021 for the largest temperate meadow in China (Hulun Buir grasslands), by harmonized Landsat 7/8 and Sentinel-2 images. The GP maps used values of 0–1 to present detailed grazing gradient information. To match widely used grazing gradients, annual GI maps with ungrazed, moderately grazed, and heavily grazed levels were generated from the GP dataset with a decision tree. The GI maps for 2015–2021 had an overall accuracy of more than 0.97 having significant correlations with the statistical data at city (r = 0.51) and county (r = 0.75) scales. They also effectively captured the GI gradients at site scale (r = 0.94). Our study proposed a monitoring approach and presented annual 10-m grazing information maps for sustainable grassland management.

## Background & Summary

Grasslands cover around 40% of the earth’s surface and provide a wide range of ecosystem services, such as food supplies, carbon sequestration, and climate mitigation^1^. Grazing is the main land use in grasslands and produces about 30% of the world’s meat supply^2^. In China, about 18 million people rely on grasslands to survive with grazing as their main source of income^3^. However, the effects of grazing activities on ecosystem services are spatially complex, which depend on grazing intensity (GI) and local environmental conditions. For example, the negative and positive effects of grazing were observed divergently in warmer and colder areas^4^. In addition, grazing may have variable effects on the structure and function of grasslands in the short and long term^5^. Despite this significant spatial and temporal variability, existing studies on the effects of grazing on grasslands have primarily focused on local scales with limited field survey data^4^. One of the main obstacles to regional grazing studies is the absence of large-scale, long-term, and high-quality grazing information maps. Without these data, it is difficult to achieve a comprehensive understanding of grazing impacts, which is necessary to develop context-dependent grazing management strategies for sustainable grassland conservation. Therefore, it is essential to improve grazing monitoring techniques and produce annual maps at regional and large scales.

GI is a general indicator used to quantify the degree of grassland utilization, also called grazing pressure^6^. It was challenging to document the spatio-temporal distribution and dynamics of GI in grasslands continuously over large regions using traditional field survey approaches^7^. Another widely used approach involves spatializing the statistical data describing livestock numbers to produce GI distribution maps^8^. This method considered only the number of livestock carried but not the impacts of grazing on grasslands^9^. Satellite observations and the Google Earth Engine (GEE) platform provide an opportunity to map the spatiotemporal patterns of GI at local, regional, and global scales using long-term multi-source remote sensing archive data and state-of-the-art cloud-computing capability^10^. Moreover, it is promising to monitor the GI considering the consequences on grasslands by satellite techniques based on the characteristics of vegetation dynamics^11^. In grassland ecosystems, the feeding behaviors of herbivores directly influence the vegetation states^12,13^. For instance, livestock grazing may remove vegetation but manures will increase vegetation vitality in the short term^14^. In the long term, vegetation compositions may shift to some extent due to grazing^15,16^, which can change the density and height of vegetation. In field surveys, the GI can be assessed by the structure and function parameters of grass (e.g., height, coverage, edible pasture, and residual biomass)^17^, which can be captured by remote sensing.

Optical images were the dominant data type used in previous studies on GI mapping, e.g., Moderate Resolution Imaging Spectroradiometer (MODIS, 250-m/500-m resolution), Landsat (30-m resolution), Sentinel-2 (10-m resolution), etc. (Table 1). Previous studies have demonstrated that temporally intensive MODIS observations can be used to detect GI by phenology features^18,19^. However, the coarse-spatial resolution of these data makes it hard to characterize the spatial heterogeneity of grazing activities. In recent years, Landsat images have been widely used in grazing studies due to their high spatial resolution (30-m) and long image archive^9,20^. For example, Landsat data were used to capture the dynamics of grazing pressure in the steppe of northern Kazakhstan from 1985 to 2017^9^. However, the 16-day time interval may be insufficient to capture the grassland dynamics caused by the behavior of free-grazing livestock^21,22^. Currently, a combined time series of Landsat and Sentinel-2 images can be used to achieve improved observations with high spatial (10-m) and temporal (≤ 10-day) resolutions, which have been widely used to monitor vegetation phenology and crop types^23,24^. However, no studies have attempted to monitor the dynamics of GI in grasslands based on the 10-m harmonized Landsat and Sentinel-2 time series.

**Table 1:** Summary of the main literature related to the monitoring of grazing intensities in grasslands.

Method	Satellite	Images used	Spatial resolution	Study period	Samples	References
Random forest (RF)	Landsat	Images from April to October of each year with 16-day time interval	30-m	1985-2017	Field data (2009, 2010, 2015, and 2016)	^9^
Convolutional neural networks (CNN)	Sentinel-2	Images of all the year round with 16-day time interval	20-m	2017, 2018	Field data (2017 and 2018)	^16^
Artificial neural network (ANN)	Landsat	Image of July 25, 2014	30-m	2014	Field data (2014)	^29^
K-means clustering	RapidEye	Images from April 24 to August 2, 2013, with about monthly time interval	5-m	2013	Visual interpretation (2013)	^48^
NDVI threshold	Landsat	Images from March 16 to September 24, 2015, with 5- and 13-day time interval	30-m	2015	Public and government data in 2015	^8^
RF	Sentinel-2	Images from August 23 to November 1, 2018, with more than a 15-day time interval	20-m	2018	Field data (2018)	^68^
Difference simulation	MODIS	Images from the day of year 97 to 225 with a 16-day time interval	250-m	2001–2014	Field data (2011, 2013 and 2014)	^69^
RF and decision-tree	Landsat + Sentinel-2	Images in the growing season identified by LST automatically with a 10-day time interval	10-m	2015–2021	Field data and visual interpretation (2015–2021)	This study

The characteristics of the previous studies are shown in the table.

Remote sensing-based algorithms for monitoring grazing intensities can be broadly categorized into difference simulation and machine learning (ML) methods. Difference simulation methods are widely used in grassland utilization intensity studies such as grazing and mowing detections^18,25^. These methods often estimate the differences between potential and actual vegetation states using various satellite-based vegetation indices. Then the differences were used as indicators to quantify the grassland utilization states. However, it is challenging to accurately estimate the potential states of vegetation using these approaches^26^. In recent years, ML methods have shown significant promise for mapping the processes of gradual change and random behaviors in land surface usage^27,28^. Several studies have successfully monitored the grazing or mowing intensity in different grassland ecosystems using various ML methods, such as Random Forest (RF) regression^9^, convolutional neural networks (CNN)^16^, and the artificial neural network (ANN)^29^. The RF method based on the probability of class members has proven to be more successful than directly using RF classifier^30,31^. Previous studies have also suggested that integrating this method and field samples with different GI gradients can detect more detailed grazing behaviors while also reducing the interference of climatic factors^9^. However, existing studies were mainly conducted based on satellite images at a specific time of the year (Table 1). Thus, it remains challenging to automatically monitor the interannual dynamics of GI over large spatial areas.

This study aimed to develop a spatial-adaptive and time-stable approach to monitor the interannual dynamics of GI at 10-m resolution at a regional scale by integrating RF classification probability and a paired GI sampling strategy based on harmonized Landsat and Sentinel-2 time series. One of the largest grasslands in China, Hulun Buir grasslands, was selected due to its complex grazing activities within a total area of 2.62 × 10^5 ^km^2^. The three objectives of the study were to (1) develop a phenology- and ML-based approach to map the grazing probability (GP) and GI automatically based on remote sensing metrics and multi-year field samples, (2) generate 10-m resolution annual GP and GI maps from 2015 to 2021 in Hulun Buir grasslands, and (3) evaluate the resultant GP and GI maps at three spatial levels (i.e., experiment sites, county, and city) using grazing experiment site and government statistical data during 2015 to 2021.

## Methods

### Study area

**Fig. 1:**
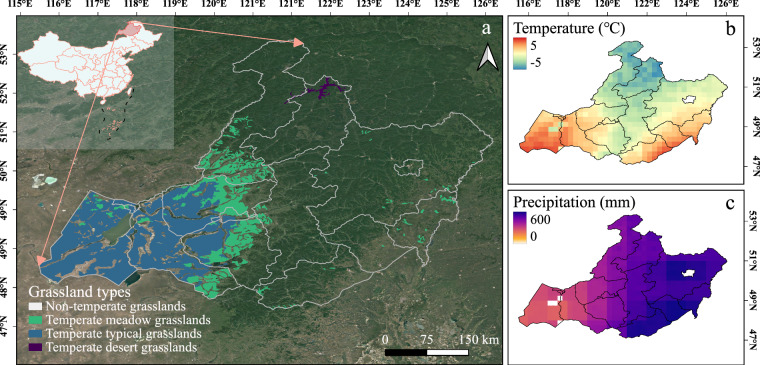
(**a**) The location and grassland types of Hulun Buir grasslands in China. (**b,c**) the distributions of mean annual temperature and mean annual precipitation from 2015 to 2021. The temperature and precipitation maps were derived from the Global Land Data Assimilation System (GLDAS) data products.

Located in the eastern part of the Eurasian steppe belt, Hulun Buir grasslands are in the northeast of Inner Mongolia (47°05′–53°20′ N, 115°31′–123°00′ E), China, with a total area of 2.62 × 10^5 ^km^2^ (Fig. 1a). It plays a vital role in the husbandry of China with intensive livestock production. It is also an important ecological barrier between Northeast China and the Beijing-Tianjin region^27^. Hulun Buir grasslands are flat and the elevation ranges from 600-m to 750-m above sea level. The study area is characterized by the temperate continental monsoon climate, which has mild and short summers, but cold and long winters. The mean annual temperature varies from −5 °C to 5 °C from 2015 to 2021 (Fig. 1b). The mean annual precipitation is around 200 mm to 600 mm from 2015 to 2021 with a decreasing trend from the northeast to the southwest (Fig. 1c). Grasslands are distributed from east to west, along with the dryness gradient of climate^22^ (Fig. 1a).

Under the influence of policy, the GI of Hulun Buir has changed in the last few years. As one of the largest pastoral areas in China, there was intensive livestock production and grassland utilization. To restore grassland ecosystems and raise herdsmen’s income, the Grassland Ecological Compensation Policy (GECP) was proposed in China in 2010, which was the largest grassland conservation program in the world. The first five-year program (GECP-I) was implemented in Hulun Buir from 2011 to 2015^32^, and the second five-year program (GECP-II) was launched in 2016^3,33^. However, how the grazing activities have been changed along with the conservation policy remains unclear in this region.

### Data

#### Landsat and Sentinel-2 data and preprocessing

We used all the Landsat-7/8 and Sentinel-2 (LC/S2) Surface Reflectance (SR) images from 2015 to 2021 at the Google Earth Engine (GEE) platform to establish the RF model. Landsat data collected at the GEE platform including all available Level-2 Landsat-7 Enhanced Thematic Mapper (ETM+) and Landsat-8 Operational Land Imager (OLI) surface reflectance data from the United States Geological Survey (USGS). Landsat data has a 30-m spatial resolution and 16-day temporal resolution. Sentinel-2 (S2) data included all available Level-2 Sentinel-2A and Sentinel-2B Multi-Spectral Instrument (MSI) data from the European Space Agency (ESA). Sentinel 2 A/B data provided 5-day interval observations at 10-m spatial resolution. The harmonized LC/S2 dataset was generated by four main steps, including excluding poor-quality observations, harmonizing LC/S2 images, producing spectral bands (SBs) and vegetation indices (VIs), and constructing time series.

Landsat images were pre-processed using the quality control layer (pixel_qa) to mask out all poor-quality observations caused by clouds, cloud shadows, snow, and scan line corrector gaps. The quality band was produced by the CFmask algorithm^34^. S2 images were also pre-processed to ensure high-quality observations. The band of cloud mask QA60 was used to identify the good observations (Gobs) on the GEE platform. Gobs were identified as the pixels without opaque and cirrus clouds. The number of Gobs and total observations were analyzed in Fig. S1. Compared with the single dataset of Landsat or Sentinel-2, the harmonized time series significantly improved the frequency of Gobs about by three times at the pixel scale. Second, the SBs from LC/S2 were harmonized using the ordinary least squares (OLS) regression^35^. Landsat 8 has improved calibration and signal-to-noise characteristics than Landsat 7^36^. We harmonized the Landsat 7 and Sentinel-2 data to the standard of the Landsat 8 data. For Landsat 7 data, we converted the values of band 1 (blue), band 2 (green), band 3 (red), band 4 (near-infrared), and band 5 (shortwave-infrared) to match Landsat 8 bands using the OLS regression coefficients^35^. For Sentinel-2 data, band 2 (blue), band 3 (green), band 4 (red), band 8 A (near-infrared), and band 11 (shortwave-infrared) were converted using another set of OLS regression coefficients^37^. Third, three VIs were calculated by the harmonized LC/S2 data, including the Normalized Difference Vegetation Index (NDVI)^38^, Enhanced Vegetation Index (EVI)^39^, and Land Surface Water Index (LSWI)^40^. NDVI and EVI are closely related to vegetation coverage, greenness, and production^41^. LSWI is a good indicator to monitor the dynamics of land surface moisture^40^. Using the surface reflectance values of blue, red, near-infrared (NIR), and shortwave-infrared (SWIR) bands from the LC/S2 images, the VIs were calculated according to the following Eqs. (1–3).

1 $$NDVI=\frac{{\rho }_{NIR}-{\rho }_{Red}}{{\rho }_{NIR}+{\rho }_{Red}}$$

2 $$EVI=2.5\times \frac{{\rho }_{NIR}-{\rho }_{Red}}{{\rho }_{NIR}+6\times {\rho }_{Red}-7.5\times {\rho }_{Blue}+1}$$

3 $$LSWI=\frac{{\rho }_{NIR}-{\rho }_{SWIR}}{{\rho }_{NIR}-{\rho }_{SWIR}}$$

Where *ρ*_*Blue*_, *ρ*_*Red*_, *ρ*_*NIR*_ and *ρ*_*SWIR*_ are the surface reflectance values of blue, red, near-infrared (NIR), and shortwave-infrared (SWIR) bands of Landsat and Sentinel-2.

Finally, because the raw LC/S2 time series have uneven observation frequency for individual pixels, we restructured the time series of SBs and VIs with a 10-day interval^24^. The NDVI and EVI were composited by calculating the maximum value within 10 days, and other bands were processed by the 10-day mean value considering the differences in land surface greenness and wetness^24^. If there were no good-quality observations in 10 days, the data gaps were filled with the linear interpolation method^42^. These processes aimed to generate consistent remote sensing data at spatial and temporal scales to reduce the uncertainties resulting from data sources.

#### MODIS Land cover data

We used the MODIS land cover product (MCD12Q1) to extract the permanent grasslands of Hulun Buir. The MCD12Q1 hosted on NASA LP DAAC at the USGS EROS Center (https://lpdaac.usgs.gov/products/mcd12q1v006), generated based on the Terra and Aqua data, provides the annual global land cover maps at 500-m spatial resolution since 2001. MCD12Q1 contains five land cover classification systems. In this study, we used the International Geosphere-Biosphere Programme (IGBP) classification scheme^43^. The annual MCD12Q1 land cover maps from 2010 to 2021 were used to extract the permanent grasslands (LC_Type1 = 10) for the study period of 2015 to 2021^42^.

#### MODIS Land surface temperature (LST) data

The MODIS land surface temperature (LST) data product level-3 (MOD11A2 and MYD11A2) from 2015 to 2021 were used to determine the nighttime LST-based thermal growing season in this study^44^. The LST products have a spatial resolution of 1 km and a temporal interval of 8 days, provided by NASA LP DAAC at the USGS EROS Center (https://lpdaac.usgs.gov/products). MOD11A2 and MYD11A2 provided the nighttime LST at ~22:30 and ~1:30 a.m. local solar time, respectively. We calculated the mean nighttime LST of MOD11A2 and MYD11A2 for the good-quality pixels based on the quality control layer.

#### Ground reference data

**Fig. 2:**
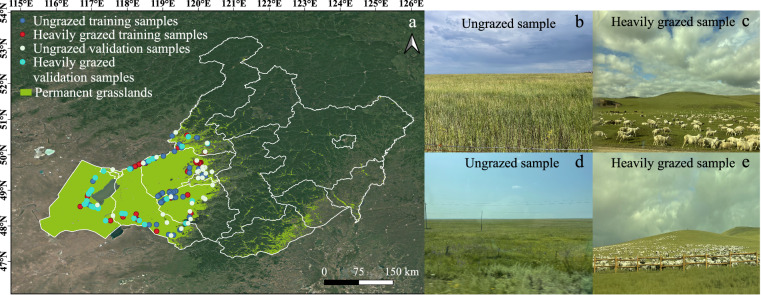
The spatial distribution of field data and permanent grasslands. (**a**) The distribution of permanent grasslands and field samples in the study area. The field samples include training samples to establish Random Forest models and validation samples for calculating a confusion matrix. (**b,d**) show the field photos of ungrazed samples. (**c,e**) show the field photos of heavily grazed samples.

Ground reference data of different GI levels are essential to generate the training and validation samples required to develop an RF model of GI mapping. To do so, more than 100 GI samples were collected in a field survey conducted in Hulun Buir from July to September 2021 (Fig. 2). These GI samples were collected from different GI levels (ungrazed and heavily grazed) by paired sampling within adjacent areas (<1 km apart). Thus, the different GI levels of adjacent samples were primarily driven by grazing activities, as neighboring plots typically had similar climates and environments^4^. In the fieldwork, samples with different GI levels were determined based on the state-of-the-art industrial standard in the *Evaluating criterion for balance of forage supply and livestock requirement*^45^. The enclosed plots without livestock grazing were selected as the ungrazed samples. Then, using the ungrazed samples as benchmarks in paired sampling, the heavily grazed samples were determined based on the reduction rates of vegetation coverage, grass height, edible forage, and residual biomass (Table 2). These evaluating criteria based on vegetation status are unaffected by grassland type and climate zone and represent a more practical approach than that based only on livestock number for grassland management.

**Table 2:** The industrial standard of national forestry and grassland administration in China for *Evaluating the balance of forage supply and livestock requirement*.

	Reduction rate of vegetation coverage (%)	Reduction rate of grass height (%)	Reduction rate of edible forage (%)	Reduction rate of residual biomass (%)
Ungrazed	Benchmark	Benchmark	Benchmark	Benchmark
Moderately grazed	11–20	11–20	11–20	11–20
Heavily grazed	>20	>20	>20	>20

This criterion was used as a reference to collect field samples with different grazing intensity levels.

*The edible forage is the plant species that can be eaten by animals except inedible or poisonous grass. The residual biomass is the quality of existing green living organic dry matter on grasslands after grazing.

The grasslands of Hulun Buir have been separated into public and private regions. The public grasslands were used for free grazing, while parts of the private grasslands were enclosed as grazing prohibition areas restricted by GECP^46^. During the field trip, we also investigated the utilization of grasslands over the past few years by the survey of herdsmen. The investigation covered the locations of public and enclosed grasslands, grazing route, number of livestock, grassland production from 2015 to 2021. Based on the field survey information, we further verified the stability of the field samples by visual interpretation of high-resolution images from Google Earth and the high-resolution earth observation system of China (GF1/GF2) from 2015 to 2021. We digitized the multi-year unchanged regions of interest (ROIs) for ungrazed and heavily grazed samples during 2015 to 2021. Then, the samples were split into training and validation groups randomly with a proportion of about 8:2 at the pixel scale. The training samples consisted of ungrazed (45 ROIs, 1.53 × 10^5^ pixels) and heavily grazed (60 ROIs, 1.97 × 10^5^ pixels) ones. The validation samples comprised ungrazed (35 ROIs, 3.61 × 10^4^ pixels) and heavily grazed (28 ROIs, 3.55 × 10^4^ pixels) ones, which were used to evaluate the accuracy of the GI maps in 2015–2020 by calculating the confusion matrixes.

## Methods

**Fig. 3:**
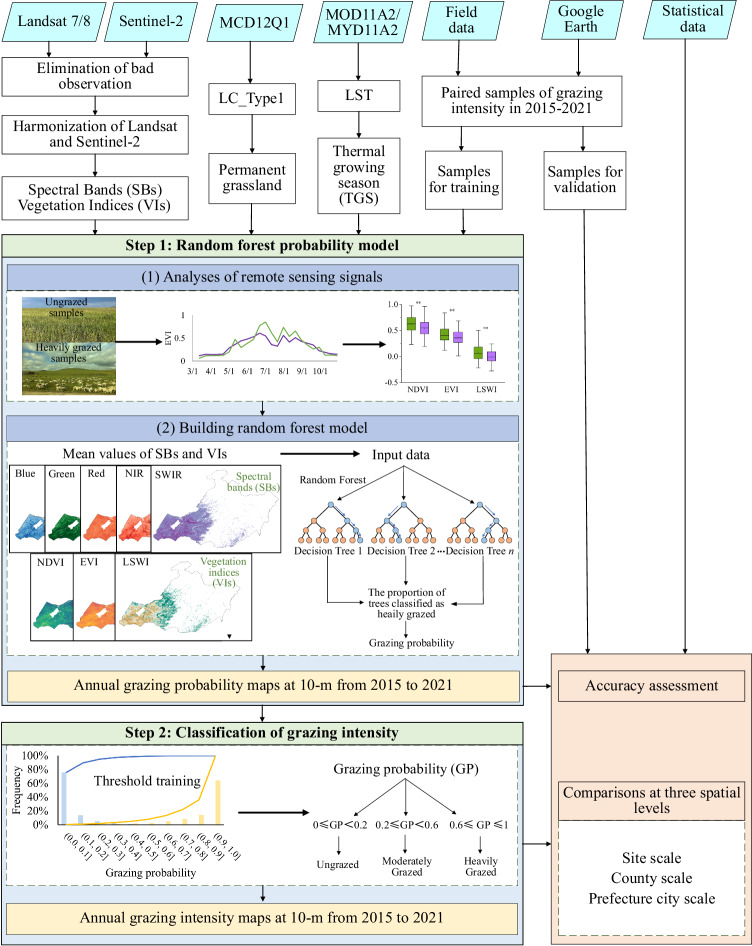
The workflow for grazing intensity mapping by combining the time series of Landsat 7/8 and Sentinel-2. It included three main sections of grazing probability mapping, grazing intensity mapping, and accuracy assessment and comparisons.

Figure 3 shows the workflow of mapping the annual GI from 2015 to 2021. There were three main sections. We first developed a phenology- and RF-based approach to producing annual GP datasets by selecting sensitive variables (SBs and VIs) based on harmonized LC/S2 images. Then, the annual 10-m GI maps were generated based on the resultant GP maps across the permanent grasslands in the study area. Finally, the resultant GP and GI maps were evaluated by accuracy assessment and cross-comparison with third-party datasets at three spatial scales from 2015 to 2021. The detailed information is described in the following text.

### Extraction of permanent grasslands

This study focused on the permanent grasslands during the study period without considering the grasslands that had land-use conversions (e.g., grassland reclamation). The permanent grasslands were extracted based on the MCD12Q1 land cover dataset from 2011 to 2021. LC_Type1 land cover had an IGBP classification scheme with a value of 10 denoting the grasslands^47^. We first identified the grassland pixels to map the annual grassland maps from 2011 to 2021. Then, the grassland frequency (GF) map was generated by the sum of the annual grassland maps. Finally, the permanent grasslands were extracted by identifying the pixels with always grassland cover (GF equal to 11) during 2011–2021. This map provided the permanent grasslands (7.63 × 10^4 ^km^2^), which was about third of all Hulun Buir grasslands, for GI analyses in the following studies.

### Automatic identification of thermal growing season at the pixel scale

Due to the limitations of temperature to vegetation productivity, the start and end of the growing season can be defined as the first day when the minimum temperature is higher and lower than 5 °C, respectively, following previous studies^48^. This rule can be realized at a pixel scale with a 1-km spatial resolution based on the nighttime LST time series derived from MOD11A2 and MYD11A2^49^. The nighttime LST was calculated as the mean of MOD11A2 (overpass time is ~22:30 l.s.t) and MYD11A2 (overpass ~1:30 am l.s.t) observations^50^. The thermal growing seasons (TGS) were defined as the period between the first day with nighttime LST above and below 5 °C for each year (2015–2021). This nighttime LST-based TGS was used to extract the metrics of SBs and VIs within the annual growing season in the following works.

### Signature analysis of different grazing intensities

There were obvious spectral differences in grasslands with different grazing intensities, which can be used to develop remote sensing-based approaches to monitor GI. SBs of LC/S2 data included blue, green, red, NIR, and SWIR. VIs included the NDVI, EVI, and LSWI, which have been widely used to study vegetation dynamics across multiple spatial scales^51^.

**Fig. 4:**
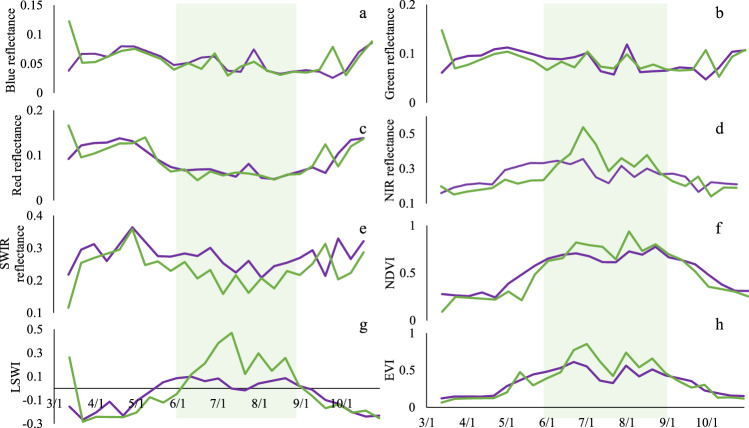
The remote sensing signatures of grasslands in different grazing intensities were analyzed based on the LC/S2 harmonized time series of 2021. A couple of paired samples in heavily grazed and ungrazed were taken as an example. (**a**–**h**) show the time series of spectral bands (blue, green, red, NIR, and SWIR) and vegetation indices (NDVI, EVI, and LSWI) in 2021. The growing season was marked with a green shadow in (**a**–**h**), identified by the start and end times of LST over 5 °C.

**Fig. 5:**
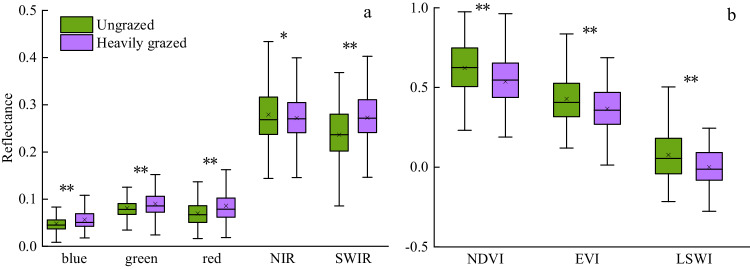
The boxplots of eight metrics for all ungrazed and heavily grazed samples. (**a**) Five spectral bands included blue, green, red, NIR, and SWIR, and (**b**) three vegetation indices included NDVI, EVI, and LSWI. The metrics with significant differences between heavily grazed and ungrazed samples were denoted as ** (P < 0.01) and * (P < 0.05), respectively.

To select the sensitive metrics to build the RF model for GI mapping, we compared the seasonal characteristics of SBs and VIs for the heavily grazed and ungrazed samples (Figs. 4, 5). Typical sample analyses showed the signatures of the harmonized LC/S2 time series within the TGS could classify the ungrazed and grazed fields with low noise interference (Fig. 4). Furthermore, a set of LC/S2-based SBs and VIs in TGS were analyzed based on the multi-year (2015–2021) training ROIs of ungrazed (45 ROIs, 1.53 × 10^5^ pixels) and heavily grazed (60 ROIs, 1.97 × 10^5^ pixels) grasslands (Fig. 5). The SBs (Fig. 5a) and VIs (Fig. 5b) of heavily grazed and ungrazed samples had significant differences with P < 0.01 except the NIR band with P < 0.05. VIs of the ungrazed samples were higher than those of the heavily grazed samples because ungrazed grasslands often have greater vegetation biomass, coverage, and moisture than the heavily grazed grasslands^52^. These analyses showed the SBs and VIs within the TGS were promising variables that can be used to indicate grazing gradients to produce annual GI maps. We used the SBs and VIs as variables to establish the RF model for GI mapping (see the following section).

### Monitoring of grazing probability based on random forest

An RF classifier was applied to quantify GP using the harmonized LC/S2 data within the TGS. According to the results of signature analyses, the mean values of five SBs and three VIs within the TGS were used as input variables to build the RF model. The RF model was trained by the multi-year reference data from 2015 to 2021. Based on the probability of the RF classifier, the GP from ungrazed to heavily grazed has been generated. Specifically, two contrasting ends of the grazing gradient, heavily grazed and ungrazed samples, were used to train the RF model based on all the pixels from the training ROIs. RF is an ensemble learning algorithm, which is more accurate and robust to noise than single algorithms^53^. The RF model was established using the scikit-learn library in Python^54^. This model calculated the mean probability by all trees for each class. We used the probabilities of the heavily grazed class to indicate the GP of grasslands. The probability shows how likely a pixel belongs to the heavily grazed class. We tuned two parameters of the RF model: (1) *n_estimators* into 1000, which was the number of trees in the forest; and (2) *max_features* into *sqrt*, which was the number of features to consider when looking for the best split. Ten-fold cross-validation was adopted to select the best RF model. This generalized model trained by multi-year samples was applied to produce the annual GP maps based on the SBs and VIs of LC/S2 from 2015 to 2021.

To reduce the salt-and-pepper noise on the resultant maps, a smoothness approach was employed to improve the accuracy, which was a commonly used method of post-classification processing, especially for high spatial resolution images^55^. We compared four widely used filters of 5 × 5 median filtering, 3 × 3 median filtering, 5 × 5 mean filtering, and 3 × 3 mean filtering using the ground reference data. At last, the 5 × 5 median filtering had the best performance that was used to generate the annual GP maps for 2015–2021.

### Classification of grazing intensity

**Fig. 6:**
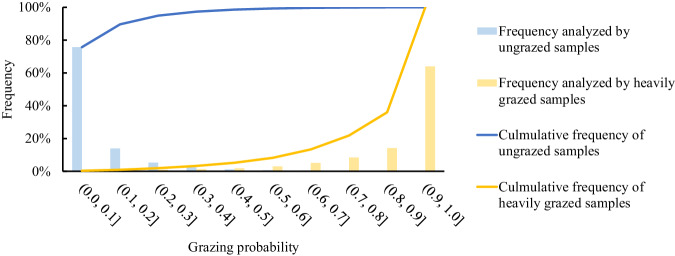
Frequency analysis of ungrazed and heavily grazed samples in the grazing probability map in 2021 using decision tree algorithm.

This approach, which used RF-derived class membership probabilities for different class types to map land use/land cover, has been widely used in previous studies of forest, cropland, and wetland mapping^28,56,57^. The potential of this approach has also been reported in the grazing pressure identification in Kazakhstan^9^. Thus, in this study, a similar method was used to estimate GI based on the resultant GP maps and ground samples from different GI levels. To determine the thresholds for different GI levels, we used the decision tree algorithm to examine the frequency distribution of the field-based heavily grazed and ungrazed samples in the GP map in 2021 (Fig. 2). To extract the majority of the information while not introducing too much noise, the thresholds were determined following the cumulative frequency (Fig. 6). A GP threshold of 0.6 was used to extract the heavily grazed pixels (values ≥0.6) in our annual maps, while the ungrazed areas were mapped with a threshold of 0.2 (values ≤0.2) (Fig. 6). Figure 6 shows that these thresholds can extract more than 90% of the information. Other pixels with GP between 0.2 and 0.6 were classed as moderately grazed areas. The decision tree algorithm used here was a data-driven model that established a relationship between GI and GP based on samples, which was then applied to GI mapping across larger regions^9^.

### Accuracy assessment and comparison

The classification accuracy of annual GI maps was evaluated by a confusion matrix. Based on the field survey and high-resolution images, the validation samples of ungrazed (35 ROIs, 3.61 × 10^4^ pixels) and heavily grazed (28 ROIs, 3.55 × 10^4^ pixels) were collected for the study area. These validation samples were used to assess the accuracy of the GI map in 2015–2021. A confusion matrix was established to calculate the overall accuracy (OA), producer’s accuracy (PA), and user’s accuracy (UA).

We compared our annual GP and intensity maps with the statistical data at two administration levels of the prefecture city and county in Hulun Buir. The annual statistical data of livestock numbers at the year-end were collected from the statistical yearbooks by Inner Mongolia for the available period of 2015 to 2020. The statistical data in 2021 have not been updated, so it was not used in this study. We collected the large livestock and small livestock numbers and converted them to standard sheep unit using the coefficients in Table 3^58^. These coefficients were the simplified coefficients because the end-year number of small and large livestock data was more abundant than that for different kinds of animals. Then the annual livestock density was calculated by the livestock number and county area. At last, we compared our resultant GP map with the annual livestock density data at the prefecture city and county scales. This cross-comparison was to evaluate the spatial-temporal agreements between the remote sensing approach and the agricultural statistic approach.

**Table 3:** The conversion coefficients of large and small livestock to standard sheep unit.

Livestock species	Standard sheep unit accounting coefficient
Large livestock (cattle, buffalos, horses, donkeys, mules, camels, etc.)	4
Small livestock (sheep, goats, and hogs, etc.)	1

*Standard sheep unit was defined as an adult sheep weighing 45 kg and consuming 1.8 kg of standard hay per day.

In addition, we compared our results with the experiment data from a grazing platform at the Hulun Buir Meadow Grassland Ecosystem Field observation and experiment station^59^. This platform has been carrying out grazing experiments on beef cattle since 2009. There were 18 grazing plots including 6 levels of GI treatments with each treatment repeating 3 times (Fig. 11). Each grazing plot has a size of 5.01 hm^2^. Thus, we can examine the spatial patterns and correlations between our GP maps and the grazing plots with different GI levels. The comparison was to evaluate whether our GP dataset can distinguish different grazing states at the site scale.

## Data Records

The GP and GI datasets for the Hulun Buir grasslands in 2015–2021^60^ were named by the year in a GeoTIFF format. The spatial resolution was 10-m. The pixel values of GP and GI maps are non-dimensional. The GI was defined following the industrial standard of national forestry and grassland administration in China (Table 2). A data description file named “data_description.doc” has been uploaded online to introduce the datasets in detail^60^.

### Annual maps of grazing probability from 2015 to 2021

**Fig. 7:**
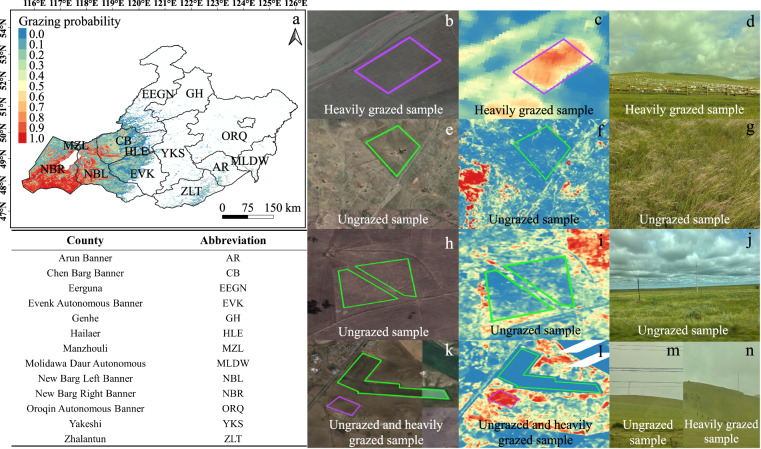
(**a**) The grazing probability map of Hulun Buir in 2021. (**b,e,h,k**) show the zoom-in views in Google Earth for samples with different grazing intensities. (**c,f,i,l**) show the zoom-in views in the grazing probability map in 2021 for the same samples as in the figures of b, e, h, and k. The purple and green polygons show the heavily grazed and ungrazed samples, respectively. (**d,g,j,m,n**) show the field photos of 2021 for the same samples as in the figures of b, e, h, and k.

**Fig. 8:**
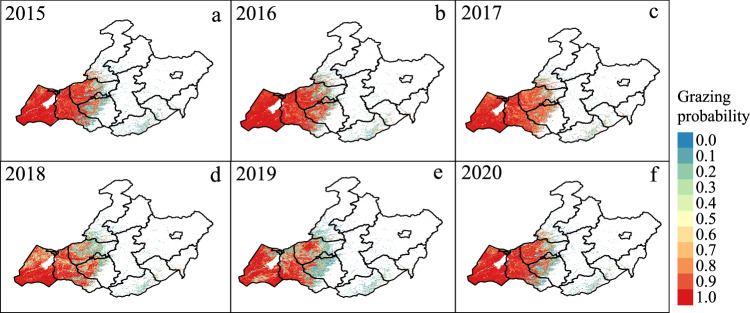
The annual grazing probability maps of Hulun Buir grasslands, China, from 2015 to 2020.

Figure 7 shows the GP map of 2021 in Hulun Buir grasslands based on the RF model developed in this study, which received a model score of 0.95 in the ten-fold cross-validation. The GP was normalized into 0~1 with the larger value denoting the higher probability under heavily grazed. Figure 7 b-n were the zoom-in views of heavily grazed and ungrazed samples in the resultant map, Google Earth images, and field photos, respectively. The results demonstrated that the GP maps have the potential to clearly distinguish different grazing states at the site scale under various grazing management activities (Fig. 7). The developed model was further used to generate the annual GP maps from 2015 to 2020 (Fig. 8). Based on the resultant maps, the spatial and temporal dynamics of GP from 2015 to 2021 were examined at the regional and county scales in Fig. S3.

### Annual maps of grazing intensity from 2015 to 2021

**Fig. 9:**
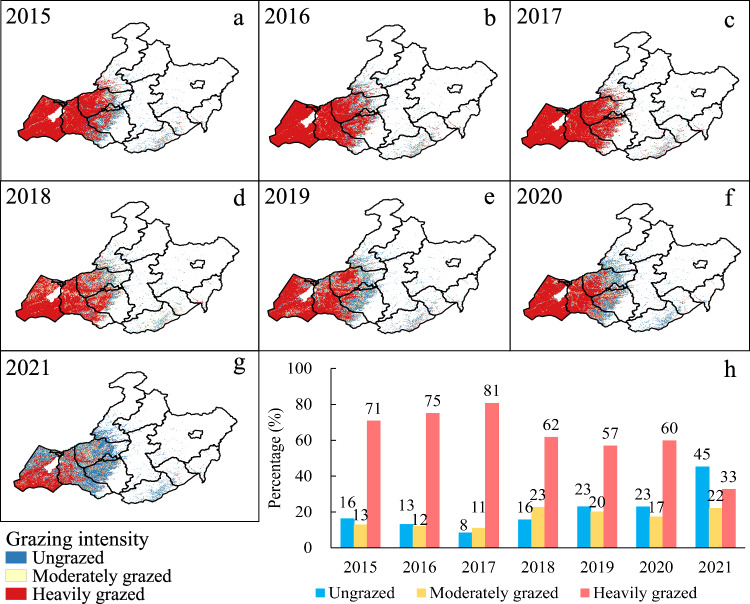
(**a**–**g**) The annual grazing intensity maps from 2015 to 2021. (**h**) Annual dynamics of proportions for each grazing intensity level (i.e., ungrazed, moderately grazed, and heavily grazed) from 2015 to 2021.

We produced the annual GI maps from 2015 to 2021 based on the resultant GP maps by decision thresholds (Fig. 9a–g). In terms of the spatial patterns, the annual GI in the west of Hulun Buir was higher than that of other regions every year (Fig. 9), which was supported by a recent study on land degradation in the study area during 2015–2021^61^. Figure 9h shows the annual dynamics of the proportions of three GI levels (i.e., ungrazed, moderately grazed, and heavily grazed levels) from 2015 to 2021. The GI in 2017 was the highest with 81% of permanent grasslands under heavily grazed (Fig. 9h). However, the trend of heavily grazed was decreasing in the study period. Meanwhile, the proportions of moderately grazed and ungrazed areas had increasing trends. These results suggested that the GI was mitigated from 2015 to 2021, despite of heavily grazed area still accounting for 33% in 2021. This result was expected due to the second phase of the grassland ecological compensation policy implemented from 2016 to 2020 to relieve the grazing pressure of grasslands^62^.

## Technical Validation

### Accuracy assessment of the annual grazing intensity maps

The accuracy of the GI maps in 2015–2021 was evaluated using the validation samples by calculating confusion matrixes including the ungrazed and heavily grazed classes (Tables S1–S7). The confusion matrixes were calculated at the pixel scale by overlaying the validation ROIs and GI maps. The results showed that the GI maps from 2015 to 2021 had the ungrazed class with the UA and PA ranging from 0.972 to 0.998 and 0.980 to 0.997, and the heavily grazed class with the UA and PA ranging from 0.978 to 0.997 and 0.975 to 0.998 (Tables S1–S7). The OA of the maps from 2015 to 2021 was between 0.985 and 0.997 (Tables S1–S7). The kappa coefficients in 2015–2020 were evaluated between 0.934 and 0.992 (Tables S1–S7).

### Comparisons of annual grazing probability maps at three spatial scales

**Fig. 10:**
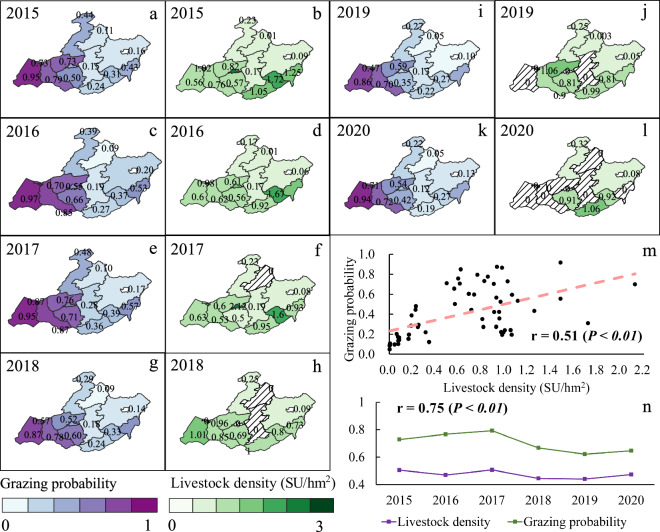
(**a**–**l**) The comparison of grazing probability maps with the livestock density. The livestock density was obtained by the ratio of livestock number at the year-end to the area of each county. The unit was standard sheep unit per hectare (SU/hm^2^). Shaded areas were counties with missing data. (**m** and **n**) The correlations of the annual mean grazing probability and the livestock density at the county and prefecture city level.

We compared the spatial and temporal dynamics of annual GP maps with the livestock data from the government at the county and prefecture city scales (Fig. 10). We obtained livestock density by the ratio of livestock number at the year-end to the area of each county. Due to the unavailable statistical data in 2021, we did not show the results for this year. At the county scale, the spatial patterns of GP maps from 2015 to 2020 were consistent with the spatial distributions of the annual livestock density. Both of them showed a decreasing pattern from west to east (Fig. 10a-l). Figure 10m shows a significant linear relationship (r = 0.51, P < 0.01) between the grazing probabilities extracted from the resultant maps and the livestock density from statistical data in 2015–2020. In some counties, the relationship was not consistent which could be caused by the high-producing breeding farm.

At the prefecture city scale of Hulun Buir, we examined the interannual dynamics of livestock density and GP from 2015 to 2020 (Fig. 10n). The results demonstrated the dynamics of livestock density and grazing probabilities agreed well with each other (r = 0.75, P < 0.01). Both variables showed decreased trends from 2015 to 2020. Subtle distinguishes in interannual fluctuations between them could be caused by the livestock that included free-grazing and house-feeding together.

**Fig. 11:**
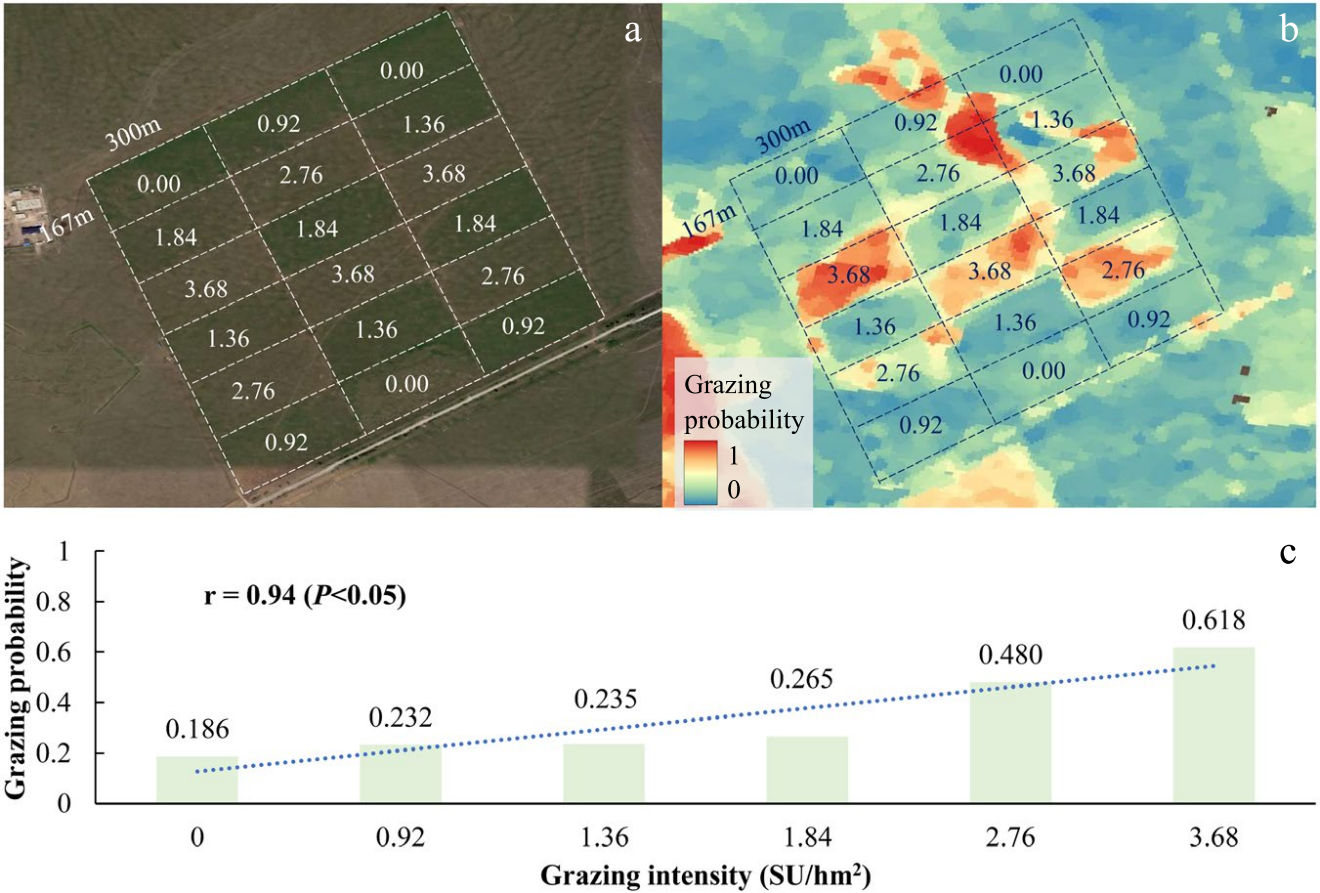
Comparisons between the resultant grazing probabilities and the grazing intensities of individual experiment plots in 2015. (**a**) shows the landscapes of the field experiment site using the high-resolution image of Google Earth. The grazing experiment has 6 levels of grazing intensity treatments of 0.00, 0.92, 1.36, 1.84, 2.76, and 3.68 per square hectare of standard sheep unit (SU/hm^2^), with each treatment repeating 3 times. (**b**) shows the result of our grazing probability map in 2015 for this experiment site. (**c**) shows the correlation between our grazing probabilities and the grazing intensity levels for the experiment plots.

In addition, we conducted a comparison with the grazing experiment data at the site scale. The grazing experiment platform had 6 levels of GI treatments with each treatment repeated 3 times (Fig. 11a). We used the GP map in 2015 as an example. The results showed that the estimated grazing probabilities from the resultant map were highly consistent with the GI levels of experiment plots (Fig. 11a,b). The correlation between the GI of experiment plots and the grazing probabilities of our results was significant (P < 0.05) with a correlation coefficient of 0.94 (Fig. 11c). With the increases of GI for individual grazing plots, the estimated grazing probabilities also increased with the maximum GP occurred at the highest GI of 3.68 per square hectare of standard sheep unit (SU/hm^2^) (Fig. 11c).

### The uncertainty of grazing probability and grazing intensity maps

The uncertainty on the annual GP and GI maps could be caused potentially by data quality and certain disturbance events. Although the Landsat archive has the potential to map the grazing intensity of grasslands, the observation quality was seriously affected by local climate conditions^63^, leading to the effective observations often limited^39^. To alleviate the uncertainty from the input images, we combined Landsat 7/8 and Sentinel-2 data to construct a time series of SBs with high observation frequency to map grazing probability and intensity. The number of good observations and total observations were analyzed in Fig. S1. Compared with a single dataset of Landsat or Sentinel-2, the harmonized time series significantly improved the frequency of good observations about by three times at the pixel scale.

Another potential uncertainty could be caused by some local disturbance events such as wildfire and wildlife, which affected the status of grassland vegetation. In this study, we analyzed the wildfire distribution from MODIS data from 2015 to 2021. The fire events in Hulun Buir were few and almost happened in the ungrown season (Fig. S2). The wildlife of Hulun Buir is almost in forest and wetland rather than grasslands according to Terrestrial Wildlife Critical Habitat List published by China National Forestry and Grassland Administration (https://www.forestry.gov.cn/search/33196). Although the wildfire and wildlife had few impacts in Hulun Buir, the disturbance events may be a source of uncertainty of the GP and GI mapping at the regional scale.

## Usage Notes

This study produced annual GP and GI maps from 2015 to 2021. A similar approach could be applied to a longer period of monitoring by harmonizing Landsat 7/8/9 images in future studies^64^. The temporal dynamics of grazing activities could be detected by the LandTrender approach based on annual GP and GI maps^65^. As well as grazing, further studies on grassland utilization and management practices, such as mowing, enclosure, and fertilization, could also be conducted in the future.

The annual 10-m GP and GI maps produced in this work provide critical regional-scale datasets for grassland research, such as the relationships between soil microbial communities and GI^66^, carbon cycle responses, and greenhouse gas emissions effects on GI^67^. The resultant maps can help the government evaluate the effects of grassland ecological compensation policy for each county and improve management approaches to achieve grassland sustainability.

### Supplementary information

Supplementary Information

## Data Availability

RF was run with scikit-learn (https://scikit-learn.org/stable/modules/generated/sklearn.ensemble.RandomForestClassifier.html) under Python 3.7. The pre-processing and harmonized code of Landsat-7/8 and Sentinle-2^60^ has been uploaded to Figshare.
