# Supplementary material for: A 10-m annual grazing intensity dataset in 2015–2021 for the largest temperate meadow steppe in China
Source: Sci Data. 2024 Feb 10;11:181. doi: 10.1038/s41597-024-03017-5 (PMC10858900; doi:10.1038/s41597-024-03017-5)
Supplement: Supplementary file 1 — Supplementary Information [file 41597_2024_3017_MOESM1_ESM.pdf]

Supplementary information for

A 10-m annual grazing intensity dataset in 2015-2021 for the largest temperate meadow steppe in China

Chuchen Chang[1], Jie Wang[1]*, Yanbo Zhao[1], Tianyu Cai[1], Jilin Yang[1,2], Geli Zhang[3], Xiaocui Wu[4], Munkhdulam Otgonbayar[5], Xiangming Xiao[6], Xiaoping Xin[7], Yingjun Zhang[1]

[1] College of Grassland Science and Technology, China Agricultural University, Beijing 100193, China

[2] Hubei Key Laboratory of Regional Ecology and Environmental Change, China University of Geosciences, Wuhan 430074, China

[3] College of Land Science and Technology, China Agricultural University, Beijing 100193, China

[4] Department of Natural Resources and Environmental Sciences, University of Illinois at Urbana-Champaign, Urbana, IL 61801, USA

[5] Division of Physical Geography and Environmental Research, Institute of Geography and Geoecology, Mongolian Academy of Sciences, Ulaanbaatar 15170, Mongolia

[6] Department of Microbiology and Plant Biology, Center for Earth Observation and Modeling, University of Oklahoma, Norman, OK 73019, USA

[7] National Field Scientific Observation and Research Station of Hulunbuir Grassland Ecosystem in Inner Mongolia, Institute of Agricultural Resources and Regional Planning, Chinese Academy of Agricultural Sciences, Beijing 100081, China

*Corresponding author: Dr. Jie Wang.
College of Grassland Science and Technology, China Agricultural University
Yuanmingyuan West Road 2, Beijing 100193, China
Email: jiewang178@cau.edu.cn

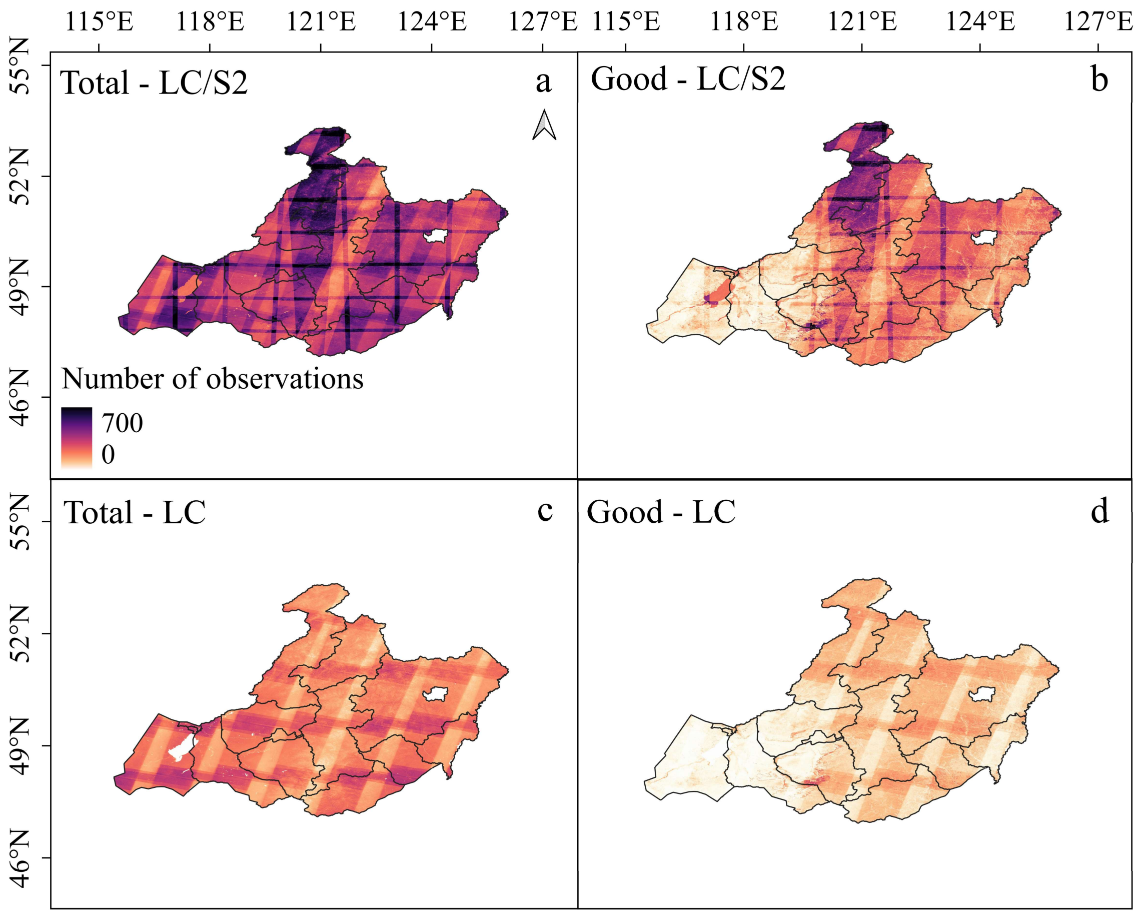

Fig. S1. Number of observations. (a) the number of total observations of harmonized Landsat 7/8 and Sentinel-2 in 2015-2021. (b) the number of good observations of harmonized Landsat 7/8 and Sentinel-2 in 2015-2021. (c) the number of total observations of only Landsat 7/8. (d) the number of good observations of only Landsat 7/8.

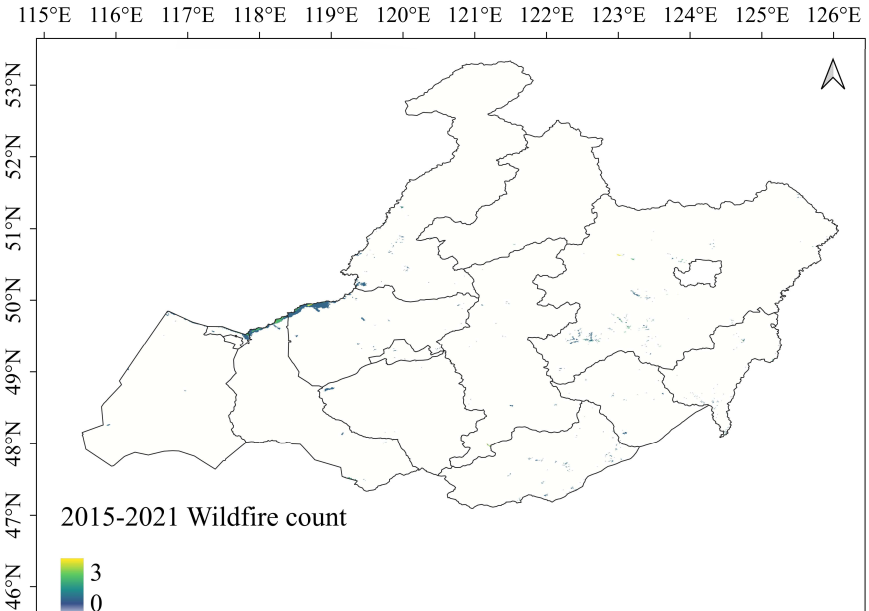

Fig. S2. The fire events in Hulun Buir happened from 2015 to 2021 based on MODIS data.

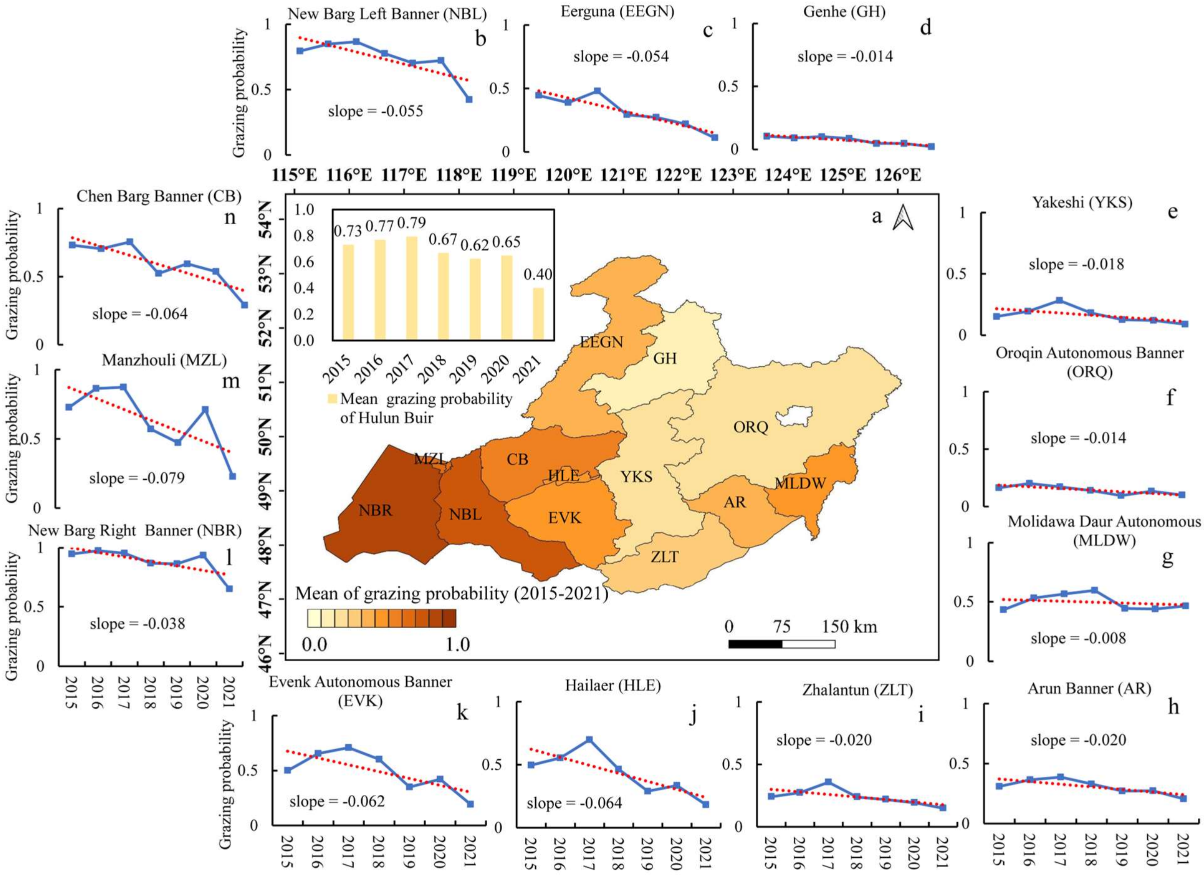

Fig. S3. Spatial and temporal dynamics of grazing probability from 2015 to 2021 at the county scale. (a) The mean grazing probability of every county from 2015 to 2021. The inset figure shows the mean annual grazing probability of Hulun Buir from 2015 to 2021. (b-n) The annual mean grazing probability and corresponding trend of each county were shown using blue dot lines and red dash lines in the figure.

Table S1. Accuracy assessment of grazing intensity map in 2021 for the ungrazed and heavily grazed types based on the validation region of interests (ROIs) from field survey and Google Earth images. The confusion matrix was calculated at the pixel scale. This table shows the User's (UA), Producer's (PA), and Overall (OA) accuracies of each grazing intensity type. The kappa coefficient in 2021 was 0.934.

		**Reference**			UA	PA	OA
		Ungrazed	Heavily grazed	Total			
	Ungrazed	3.54×10^4	737	3.62×10^4	0.998	0.980	
**Map**	Heavily grazed	88	3.26×10^4	3.27×10^4	0.978	0.997	
	Total	3.55×10^4	3.34×10^4	6.89×10^4			0.988

Table S2. Accuracy assessment of grazing intensity map in 2020 for the ungrazed and heavily grazed types based on the validation region of interests (ROIs) from field survey and Google Earth images. The confusion matrix was calculated at the pixel scale. This table shows the User's (UA), Producer's (PA), and Overall (OA) accuracies of each grazing intensity type. The kappa coefficient in 2020 was 0.989.

		**Reference**			UA	PA	OA
		Ungrazed	Heavily grazed	Total			
	Ungrazed	3.55×10^4	684	3.62×10^4	0.998	0.981	
**Map**	Heavily grazed	62	3.44×10^4	3.44×10^4	0.980	0.998	
	Total	3.55×10^4	3.51×10^4	7.06×10^4			0.989

Table S3. Accuracy assessment of grazing intensity map in 2019 for the ungrazed and heavily grazed types based on the validation region of interests (ROIs) from field survey and Google Earth images. The confusion matrix was calculated at the pixel scale. This table shows the User's (UA), Producer's (PA), and Overall (OA) accuracies of each grazing intensity type. The kappa coefficient in 2019 was 0.990.

		**Reference**			UA	PA	OA
		Ungrazed	Heavily grazed	Total			
	Ungrazed	3.57×10^4	222	3.60×10^4	0.998	0.994	
**Map**	Heavily grazed	62	3.48×10^4	3.48×10^4	0.994	0.998	
	Total	3.59×10^4	3.50×10^4	7.08×10^4			0.996

Table S4. Accuracy assessment of grazing intensity map in 2018 for the ungrazed and heavily grazed types based on the validation region of interests (ROIs) from field survey and Google Earth images. The confusion matrix was calculated at the pixel scale. This table shows the User's (UA), Producer's (PA), and Overall (OA) accuracies of each grazing intensity type. The kappa coefficient in 2018 was 0.991.

		**Reference**			UA	PA	OA
		Ungrazed	Heavily grazed	Total			
	Ungrazed	3.34×10^4	146	3.36×10^4	0.998	0.996	
**Map**	Heavily grazed	76	3.50×10^4	3.51×10^4	0.996	0.998	
	Total	3.35×10^4	3.51×10^4	6.86×10^4			0.997

Table S5. Accuracy assessment of grazing intensity map in 2017 for the ungrazed and heavily grazed types based on the validation region of interests (ROIs) from field survey and Google Earth images. The confusion matrix was calculated at the pixel scale. This table shows the User's (UA), Producer's (PA), and Overall (OA) accuracies of each grazing intensity type. The kappa coefficient in 2017 was 0.992.

		Reference			UA	PA	OA
		Ungrazed	Heavily grazed	Total			
	Ungrazed	3.29×10^4	131	3.30×10^4	0.973	0.996	
**Map**	Heavily grazed	904	3.53×10^4	3.62×10^4	0.996	0.975	
	Total	3.38×10^4	3.54×10^4	6.92×10^4			0.985

Table S6. Accuracy assessment of grazing intensity map in 2016 for the ungrazed and heavily grazed types based on the validation region of interests (ROIs) from field survey and Google Earth images. The confusion matrix was calculated at the pixel scale. This table shows the User's (UA), Producer's (PA), and Overall (OA) accuracies of each grazing intensity type. The kappa coefficient in 2016 was 0.977.

		Reference			UA	PA	OA
		Ungrazed	Heavily grazed	Total			
	Ungrazed	3.20×10^4	100	3.21×10^4	0.972	0.997	
**Map**	Heavily grazed	904	3.52×10^4	3.61×10^4	0.997	0.975	
	Total	3.29×10^4	3.53×10^4	6.82×10^4			0.985

Table S7. Accuracy assessment of grazing intensity map in 2015 for the ungrazed and heavily grazed types based on the validation region of interests (ROIs) from field survey and Google Earth images. The confusion matrix was calculated at the pixel scale. This table shows the User's (UA), Producer's (PA), and Overall (OA) accuracies of each grazing intensity type. The kappa coefficient in 2015 was 0.985.

		Reference			UA	PA	OA
		Ungrazed	Heavily grazed	Total			
	Ungrazed	3.26×10^4	293	3.29×10^4	0.980	0.991	
**Map**	Heavily grazed	659	3.51×10^4	3.58×10^4	0.992	0.982	
	Total	3.33×10^4	3.54×10^4	6.87×10^4			0.986
